# Unraveling the intricacies of typical atrial flutter

**DOI:** 10.1016/j.hrcr.2024.04.003

**Published:** 2024-06-17

**Authors:** Margaret Harvey

**Affiliations:** Department of Acute and Tertiary Care, College of Nursing, University of Tennessee Health Science Center, Memphis, Tennessee

## Introduction

Atrial flutter (AFL) is classified as either “typical” or “atypical.” Most cases are typical, so it is important to recognize associated electrocardiographic (ECG) features, conduction patterns, and how it is distinguished from atypical AFL. This case report highlights a patient with characteristic ECG findings of typical AFL who underwent a successful radiofrequency ablation.

## Case report

**Figure 1:**
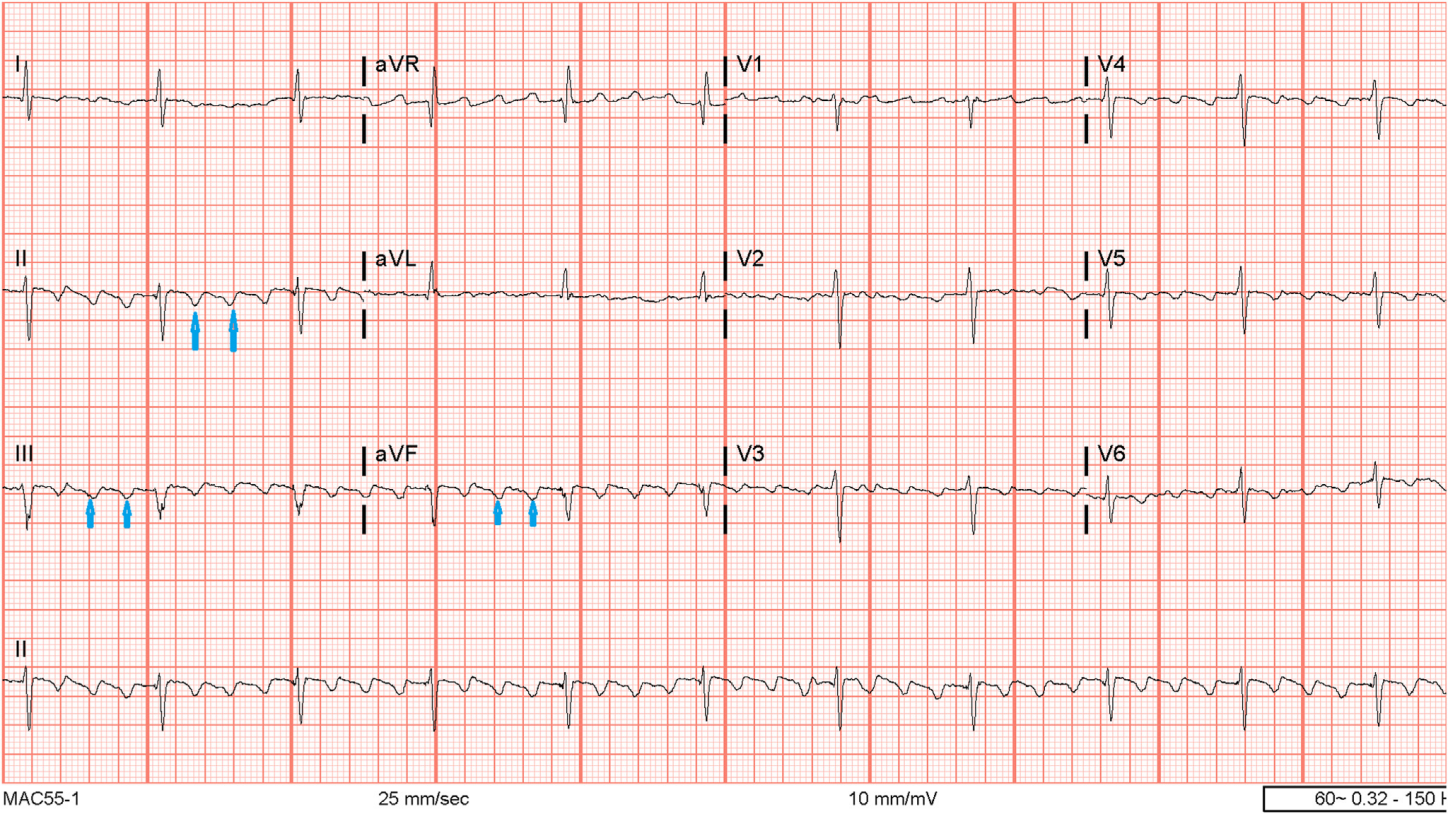
Twelve-lead electrocardiogram with sawtooth pattern flutter waves, negatively deflected in the inferior leads.

The patient is a 64-year-old man with a history of hypertension, gastroesophageal reflux disease, chronic hepatitis C, and hyperlipidemia who was admitted to the hospital for AFL with rapid ventricular response observed after a colonoscopy. The initial 12-lead ECG showed AFL with 2:1 atrial ventricular conduction and negative flutter waves in the inferior leads consistent with typical AFL (Figure 1). Transthoracic echocardiogram showed preserved left ventricular systolic function with normal atrial sizes and no evidence of intracardiac thrombus. The patient was placed on a diltiazem drip, beta-blocker, and heparin infusion with plans for radiofrequency ablation based upon a shared decision. The patient underwent an electrophysiology study and mapping that revealed a counterclockwise AFL of cycle length 240 ms, with successful cavotricuspid isthmus (CTI) ablation with termination of AFL and subsequent achievement of complete bidirectional CTI block.

## Discussion

Historically, AFL has been classified based on the ECG. Typical AFL was considered “common” when the flutter waves were predominately negative in the inferior leads with a sawtooth pattern and “atypical” when the ECG morphology differed from the common type.^1^ Classification of AFL has evolved to include anatomic substrates and mechanisms identified through activation mapping during electrophysiologic (EP) studies and is no longer referred to as type 1 and type 2.^2^

Typical AFL is the most common form and is also referred to as “CTI-dependent” AFL, whereby a macroreentrant circuit involving the tricuspid annulus traverses the CTI on the right side of the heart, ascends the atrial septum, and descends to the right atrial free wall.^3^ The direction of this circuit when viewed in the left anterior oblique fluoroscopic perspective has a counterclockwise reentry and is responsible for 90% of cases. When the activation direction is opposite, it is called “reverse typical” AFL (clockwise) and occurs in 10% of clinical cases. ECG patterns associated with counterclockwise activation include the characteristic “sawtooth pattern” with mostly negative flutter waves, whereby clockwise activation may be seen as broad, positive atrial deflections in the inferior leads.^2^

Atypical AFL is less common and involves a different circuit than the tricuspid/isthmus, also known as “non–cavotricuspid isthmus–dependent macroreentrant” AFL. Atypical AFL is often associated with structural heart disease, cardiac surgical intervention with resultant scarring, and extensive catheter ablation for atrial fibrillation.^3^ In both typical and atypical AFL, the ECG may be indistinguishable, such that an EP study with mapping is the only way to determine the mechanism and precise diagnosis.

## Conclusion

In the presenting case, the patient had ECG findings suggesting the most common form of AFL with negative flutter waves in the inferior leads. Typical AFL was confirmed during the EP study, where mapping resulted in the final diagnosis of CTI-dependent AFL, with subsequent successful radiofrequency ablation and termination of the arrhythmia. Given the intricacies of AFL, EP studies and mapping unravel the mechanism to support a precise diagnosis.

## Disclosures

The author has no conflicts of interest to disclose.
